# Considering the Influence of Nonadaptive Evolution on Primate Color Vision

**DOI:** 10.1371/journal.pone.0149664

**Published:** 2016-03-09

**Authors:** Rachel L. Jacobs, Brenda J. Bradley

**Affiliations:** 1 Department of Anthropology, Center for the Advanced Study of Human Paleobiology, The George Washington University, Washington, District of Columbia, United States of America; 2 Interdepartmental Doctoral Program in Anthropological Sciences, Stony Brook University, Stony Brook, New York, United States of America; 3 Centre ValBio Research Station, Ranomafana, Fianarantsoa, Madagascar; University of Sussex, UNITED KINGDOM

## Abstract

Color vision in primates is variable across species, and it represents a rare trait in which the genetic mechanisms underlying phenotypic variation are fairly well-understood. Research on primate color vision has largely focused on adaptive explanations for observed variation, but it remains unclear why some species have trichromatic or polymorphic color vision while others are red-green color blind. Lemurs, in particular, are highly variable. While some species are polymorphic, many closely-related species are strictly dichromatic. We provide the first characterization of color vision in a wild population of red-bellied lemurs (*Eulemur rubriventer*, Ranomafana National Park, Madagascar) with a sample size (87 individuals; *N*_X chromosomes_ = 134) large enough to detect even rare variants (0.95 probability of detection at ≥ 3% frequency). By sequencing exon 5 of the X-linked opsin gene we identified opsin spectral sensitivity based on known diagnostic sites and found this population to be dichromatic and monomorphic for a long wavelength allele. Apparent fixation of this long allele is in contrast to previously published accounts of *Eulemur* species, which exhibit either polymorphic color vision or only the medium wavelength opsin. This unexpected result may represent loss of color vision variation, which could occur through selective processes and/or genetic drift (e.g., genetic bottleneck). To indirectly assess the latter scenario, we genotyped 55 adult red-bellied lemurs at seven variable microsatellite loci and used heterozygosity excess and *M*-ratio tests to assess if this population may have experienced a recent genetic bottleneck. Results of heterozygosity excess but not *M*-ratio tests suggest a bottleneck might have occurred in this red-bellied lemur population. Therefore, while selection may also play a role, the unique color vision observed in this population might have been influenced by a recent genetic bottleneck. These results emphasize the need to consider adaptive and nonadaptive mechanisms of color vision evolution in primates.

## Introduction

Primate color vision is among the most oft-cited examples of adaptive molecular evolution [1]. It represents one of few primate traits in which variation in individual phenotypes (i.e., the ability to make or not make particular color discriminations) can be tied to small changes in single genes [2–4]. For example, shifts in the sensitivities of M/L (medium/long wavelength) cone photopigments (i.e., M/L opsins) result from just a few amino acid changes on the X-linked opsin-coding gene (M/L opsin gene) [2–4]. Accordingly, differences in color vision capacity have occurred either through gene duplication and subsequent differentiation or allelic variation of a single M/L opsin gene [2, 3, 5, 6]. The former mechanism is documented in Old World monkeys, apes, and humans, as well New World howling monkeys, and results in virtually all individuals having full trichromatic color vision [2, 5, 6]. Most other New World monkeys, on the other hand, have one M/L opsin gene with two or more alleles resulting in polymorphic color vision; heterozygous females are trichromatic, while hemizygous males and homozygous females are red-green color blind (i.e., dichromatic) ([3, 7, 8], reviews in [1, 9]). A monomorphic M/L opsin gene and dichromatic color vision appears to characterize tarsiers and some lemurs [10, 11].

Many theoretical studies suggest that these differences in color vision capacity are likely to influence specific fitness-related behaviors (e.g., foraging, predator detection) and ultimately account for observed color vision variation in primates [12–20]. For example, trichromatic color vision is thought to be advantageous for foraging on reddish food items, such as ripe fruit and/or young leaves, among a background of green foliage [13–19]. Red-green color blindness, on the other hand, may offer an advantage in detecting camouflaged objects, including some food items (e.g., insects, green fruit) and predators (e.g., snakes) [15, 21–24]. Such hypotheses are attractive, particularly because they are intuitive and also testable given that many primate species have polymorphic color vision. Accordingly, much research has been aimed at identifying how differences in color vision might be adaptive under the assumption that observed variation is shaped by selection [23, 25–31]. That said, there is mixed evidence for adaptive advantages of different color vision phenotypes in wild populations [23, 25–27, 29], with few studies testing the null hypothesis of nonadaptive evolution shaping opsin variation in primates [32].

Lemurs represent an interesting lineage to examine evolutionary mechanisms (both adaptive and nonadaptive) underlying differences in color vision. Until relatively recently, it was thought that all lemurs were either completely color blind or strictly dichromatic with a single, non-variable M/L opsin gene [33]. It is now known that some species exhibit allelic variation of the M/L opsin gene (M and L alleles) and have polymorphic color vision [11, 34–37]. In light of such studies, it appears that lemurs have more variation in color vision capacity compared to other primate lineages; monochromacy, dichromacy, and polymorphic color vision have each been documented in multiple species [11, 36–40]. At the same time, lemurs vary widely in a number of ecological characteristics, including activity pattern (nocturnality, diurnality, and cathemerality–day-and night-active), diet (e.g., gummivory, folivory, frugivory), and habitat (e.g., spiny forest, deciduous dry forest, rainforest) [41]. Each of these features has been hypothesized to influence color vision evolution [13, 39–43], and such differences are particularly relevant because color vision has been shown to vary among closely related taxa [11, 36, 37].

Within the genus *Eulemur*, for example, polymorphic trichromacy has been identified in captive *Eulemur flavifrons* [37], but other species for which published data are available (*E*. *collaris*, *E*. *mongoz*, and *E*. *fulvus*) appear to have dichromatic color vision, with only a single M opsin variant [11, 36, 43]. Species within this genus exhibit gross similarities in some ecological characteristics, being generally described as cathemeral and predominantly frugivorous [41]. However, the habitats in which *Eulemur* species live range from dry deciduous forest to rainforest [41]. Species also differ markedly in their pelage coloration and patterns, and most exhibit some degree of sexual dichromatism in coat color [41]. *Eulemur* therefore represents an ideal taxon for identifying selective pressures potentially shaping variation in color vision. Indeed, such differences in ecology, including habitat as well as potential subtle variation in activity patterns and diets, have been hypothesized to account for color vision variation among these closely related species [43].

The current published characterizations of color vision in *Eulemur* species are based on samples from captive individuals and a single wild population [11, 36, 37, 43] with sample sizes (*N*_X chromosomes_ = 4–36) below that needed (*N*_X chromosomes_ ≥ 59) to detect a low frequency allele (i.e., 0.95 probability of detecting an allele at 5% frequency based on cumulative binomial probabilities). That is, differentiating between an all-color blind population and a polymorphic population with variants at low frequencies requires greater sampling. Furthermore, in the case of captive populations (*E*. *flavifrons*, *E*. *collaris*, and *E*. *mongoz*) [11, 36, 37], it is unknown if documented M/L opsin allele frequencies are an accurate representation of those in wild populations or potentially result from founder effects.

The influence of genetic drift may also be particularly relevant to understanding genetic variation in natural lemur populations. Many studies have identified genetic signatures of historical, large-scale (in some cases orders of magnitude) population collapse in lemur species/populations across Madagascar [44–48]. Such results suggest that observed M/L opsin allele frequencies in lemurs may have been influenced by recent genetic bottlenecks and drift.

**Fig 1:**
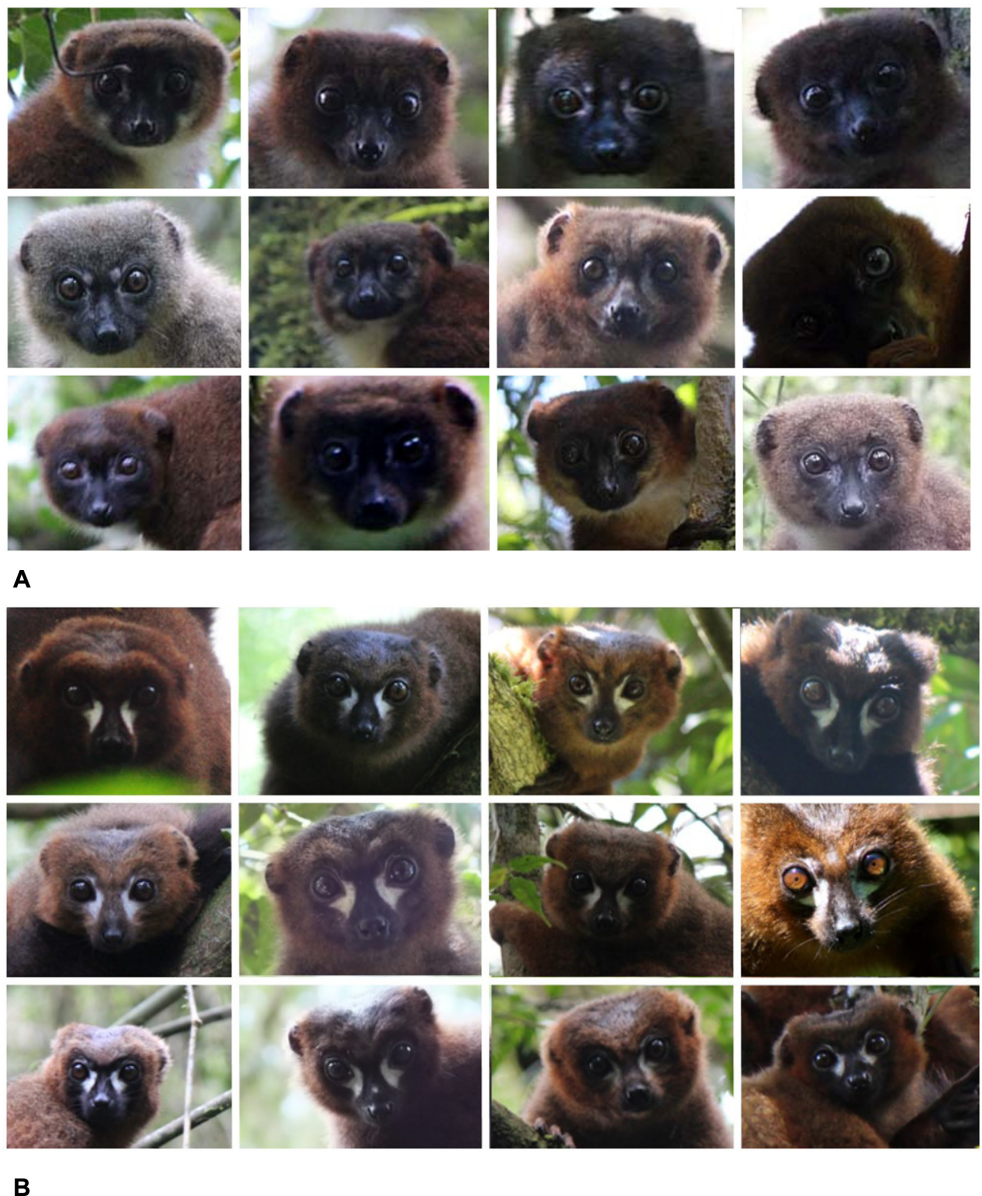
Frontal facial photographs of red-bellied lemurs in Ranomafana National Park. (A) Adult females. (B) Adult males. Photographs illustrate individual variation in facial pelage patterns used to identify individuals during data collection. Photo credits: Falinomenjanahary J., Lahitsara J.P, RLJ, and Velontsara J.B.

Thus, in order to avoid falsely inferring adaptive evolution, it is important to first explore the potential influence of nonadaptive mechanisms on present genetic variation. In this study, we characterize color vision in a wild population of red-bellied lemurs (*Eulemur rubriventer*) for which we also have detailed data on individual pelage/facial pigment variation (Fig 1) and foraging behavior. We examined coding variation in color vision (i.e., opsin genes) using an adequate sample to detect rare alleles (0.95 probability of detection at ≥ 3% frequency). We also characterized neutral genetic polymorphisms (i.e., microsatellite genotypes), which provide a baseline for understanding if the present genetic variation in this population may have been influenced by a recent genetic bottleneck.

## Materials and Methods

All methods were in compliance with and approved by Stony Brook University’s IACUC committee (IACUC #: 2010/1803, 2011/1895) and the government of Madagascar (permit #: 284/10, 157/11, 204/12, 056/13).

### Study subjects and site

**Fig 2:**
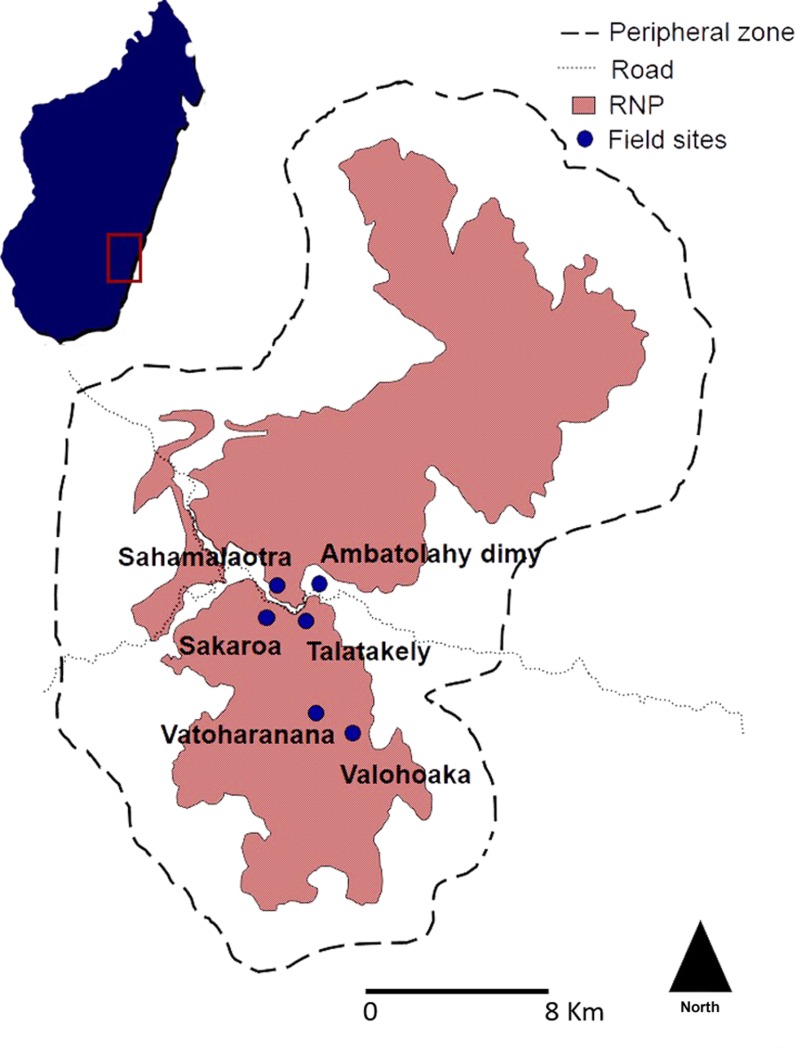
Study sites within and around RNP. Reprinted with modifications from [56] under a CC BY license, with permission from Andrea Baden, original copyright 2011.

Red-bellied lemurs live in small, cohesive groups, ranging in size from 2–6 individuals ([49, 50], this study). Adults are usually pair-living, with groups generally composed of one adult male, one adult female, and immature individuals [51]. This study was conducted on a single population of *E*. *rubriventer* in Ranomafana National Park (RNP), which is an area of 41,000 ha of montane rainforest in southeastern Madagascar (E47°18'–47°37', S21°02'–21°25') [52]. Data were collected intermittently between January 2011 and May 2013 at five site localities within the park (Talatakely, Vatoharanana, Valohoaka, Sakaroa, and Sahamalaotra), and one site located just outside the park (Ambatolahy dimy) (Fig 2). All sites are within 8 km of each other and assumed to be in migratory contact. This population of red-bellied lemurs has been the subject of previous research projects [53–55] and is exposed to ecotourism activities to varying degrees, making many individuals habituated to observer presence.

### Fecal sample collection

Fresh fecal samples for genetic analyses were collected by RLJ and field assistants from 157 individual red-bellied lemurs opportunistically as part of behavioral data collection and/or survey of the red-bellied lemur population in RNP (S1 Table). Full-day group follows (time of group location through sunset) were conducted on red-bellied lemur groups that were located by searching within known home ranges or opportunistically. During surveys, groups were visited monthly to collect demographic data. More detailed data on feeding behavior were collected on nine groups as part of related studies on color vision in this taxon. During group follows, fecal samples were collected from individual lemurs with the aim to collect three independent fecal samples/individual. All samples were collected from the ground immediately following defecation. Each sample (~ 5 grams of wet weight) was placed directly into a 50 mL plastic centrifuge tube pre-filled with 30 mL silica gel beads (for desiccation) using latex gloves and the untouched end of a freshly broken twig [57]. Tubes were labeled and sealed with parafilm and stored at ambient temperature in the field and later at +4°C in the lab.

### DNA extraction

Genomic DNA was extracted from dry fecal samples using the QIAamp^®^ DNA Stool Mini Kit (Qiagen) following the manufacturer’s protocol plus an initial 48 hour lyses in ASL buffer at room temperature. Extractions were automated using a QIAcube^®^ (Qiagen). Extraction protocols and all down-stream procedures always included negative controls.

### DNA quantitation

DNA concentrations were quantified in duplicate for all samples using a Qubit^®^ 2.0 Fluorometer (Invitrogen™) and the Qubit^®^ dsDNA HS Assay Kit. As this method quantifies total genomic DNA (including plant and microbial DNA), we also quantified samples using a real-time quantitative polymerase chain reaction (qPCR) assay targeting a conserved region of the *c-myc* proto-oncogene in primates (i.e., not amplifying non-primate DNA in samples, such as bacterial DNA) following [58]. Reactions were carried out on a Rotor-Gene Q platform using SYBR Green RT-PCR Master Mix (Qiagen). DNA quantity scores were an average of two replicates (as in [58]), and results across replicates were generally consistent.

### Sex-typing

Each sample was genetically sex-typed by amplifying segments of the tetratricopeptide repeat protein gene on the Y chromosome (UTY), and the X-chromosomal homolog (UTX) using a multiplex (triple primer) PCR following [59]. If a sample yielded both X and Y fragments, the individual was typed male, and if a sample yielded only the X fragment, the individual was typed female. Sex-typing was based on multiple independent reactions.

### M/L opsin genotyping

The opsin gene complement of individual red-bellied lemurs was determined by amplifying and sequencing a ~200 bp fragment of exon 5 of the X-linked M/L opsin gene. In platyrrhines, functional variation in M/L opsins results from amino acid site changes in both exons 3 and 5 [4]. This study focuses on the latter because functional variation in most lemur species is tied to site 285 in exon 5 [11]. Fragments were amplified using PCR with our forward primer (5’–GTAGCAAAGCAGCAGAAAGA– 3’) and a previously published reverse primer (5’–CTGCCGGTTCATAAAGACGTAGATAAT– 3’ [34]). PCR reactions were performed in 25 *μ*l total volume and contained the Qiagen HotStarTaq Master Mix, 1.6 *μ*M of bovine serum albumin, 0.4 *μ*M each of forward and reverse primers, and included 3–5 *μ*l of total template DNA. Cycling conditions were 95°C for 15 minutes and 36 cycles of 94°C for 30 seconds, 57°C for 40 seconds, and 72°C for 1 minute, with a final extension step of 72°C for 7 minutes. PCR fragments were visualized on 2% agarose gels using GelRed, and were sequenced (Sanger) in both directions using an Applied Biosystems 3730*xl* DNA Genetic Analyzer at the Yale DNA Analysis Facility. Sequence traces were scored by eye and color vision status was based on genotypes at amino acid site 285 (codon translations: GCC = alanine; ACC = threonine). All individuals were replicated 1–3 times using independent PCR reactions and 1–2 independent fecal extractions.

### Microsatellite genotyping

Samples were amplified at seven variable, unlinked microsatellite loci using previously published primers (S2 Table; [60]). Loci were selected for short fragment lengths, simple repeat motifs, and high allelic variability based on [60]. PCR conditions were carried out in 12.5 *μ*l total volume containing the Qiagen HotStarTaq Master Mix, 3.2 *μ*M bovine serum albumin, 0.8 *μ*M each of forward (fluorescently labeled) and reverse primers, and 2 *μ*l of total template DNA. DNA template volume was adjusted based on DNA quantification (>25 pg) to minimize errors associated with allelic dropout [58]. Cycling conditions were 95°C for 15 minutes and 37 cycles of 95°C for 30 seconds, a locus-specific annealing temperature (S2 Table) for 30 seconds, and 72°C for 30 seconds, with a final extension step of 72°C for 10 minutes.

Fragment length analyses were carried out in the DNA Analysis Facility at Yale University via capillary electrophoresis with an ABI 3730*xl* 96-Capillary Genetic Analyzer. Genotypes were binned and scored by eye via GeneMapper^®^ (Applied Biosystems) and GeneMarker^®^ (SoftGenetics). Homozygous genotypes were confirmed with a minimum of four and up to seven independent replications, based on results of DNA quantitation [58]. Heterozygous individuals were confirmed when each allele was scored at least twice based on two or more independent PCR reactions [58, 61].

Microsatellite genotypes were screened for errors (i.e., scoring errors, allelic dropout, and null alleles) prior to data analysis using the software MICRO-CHECKER [62]. Genepop version 4.2 was used to test for linkage disequilibrium among all combinations of microsatellite loci using the log-likelihood ratio statistic and evaluated with 10,000 permutations [63, 64]. Summary statistics for each locus (e.g., the number of alleles—*k*, number of individuals typed, observed and expected heterozygosity, and polymorphic information criterion), as well as goodness-of-fit tests for Hardy-Weinberg equilibrium were calculated in CERVUS 3.0 [65, 66]. Allelic richness (the number of alleles per locus independent of sample size) for each locus was calculated using FSTAT version 2.9.3.2.

### Genetic bottleneck analyses

#### Heterozygosity excess

The program BOTTLENECK was used to test for heterozygosity excess as a potential signal for a recent genetic bottleneck [67–69]. The program computes expected heterozygosity (H_eq_) at mutation-drift equilibrium (based on allele number and sample size) for each locus under three mutation models: infinite allele model (IAM), stepwise mutation model (SMM), and the two-phase model (TPM) [68]. The program compares H_eq_ to Hardy-Weinberg heterozygosity (H_e_) with the expectation that in recently bottlenecked populations, there will be significant excess H_e_ compared to H_eq_, because allele number should be reduced faster than heterozygosity [67, 69].

BOTTLENECK performs multiple tests, but the Wilcoxon test is considered to be robust when using a small number of polymorphic loci (< 20) and most appropriate for microsatellite data [69], and therefore is used in this study. H_eq_ is calculated under TPM, as this mutation model is also considered the most appropriate model for microsatellite loci, with IAM and SMM representing more extreme mutation models [69, 70].

Importantly, tests for heterozygosity excess have the potential to produce both type I and type II errors based in part on incorrect assumptions of mutation model parameters [71]. Specifically, TPM assumes that mutations during microsatellite evolution can result in small changes in a single repeat motif (i.e., single-step mutations, which characterize most mutations), as well as larger changes in multiple repeat motifs (i.e., multi-step mutations, which characterize fewer mutations) [70, 71]. Consequently, TPM requires knowledge (or assumptions) about the proportion and size of multi-step mutations in the microsatellite data of interest [71]. The program BOTTLENECK requires the proportion of multi-step mutations and the variance in the mean size of multi-step mutations to be specified [69], and it has been shown that type I and type II errors can result from errors in assumed values for these parameters [71, 72]. One way to help avoid such errors is to use reasonable and appropriate values for the mutation model parameters [71]. A review of 18 studies of microsatellite evolution in vertebrates suggests 0.22 and 12 are appropriate values for the proportion of multi-step mutations and variance in mean size of multi-step mutations, respectively [71]. The former value deviates from the more commonly used proportion of 0.10 [71]. Because overestimating this value increases the likelihood of a type I error in heterozygosity excess tests, 0.10 may be considered a more conservative value [71, 72]. Therefore, the Wilcoxon test was run twice under the TPM and setting the proportion of multi-step mutations to 0.22 and 0.10, respectively, with a variance of 12 for each analysis. Significance (*p* < 0.05) was assessed using 10,000 iterations.

#### *M*-ratio

A signature of a population bottleneck was also assessed using the *M*-ratio test implemented in the program M_P_val [73]. This test computes *M*, which is the ratio of *k* (total number of alleles) to *r* (range in allele size) averaged across all microsatellite loci, and compares this ratio to a simulated distribution of *M* values at mutation-drift equilibrium [73]. In populations that have experienced a bottleneck, the expectation is that observed *M* should be lower than *M* values at equilibrium, because rare alleles are likely to be lost in bottlenecked populations but should not be biased toward the smallest or largest allele sizes [73]. Therefore, *k* is expected to reduce faster than *r* [73].

Calculating *M* requires three input parameters and, similar to heterozygosity excess tests, incorrect assumptions about these parameters can produce both type I and type II errors [71]. *M*-ratio tests require assumptions about *p*_*s*_ (the proportion of one-step mutations) and *∆*_*g*_ (the average size of one-step mutations) [73]. Following the recommendation in [71] and similar to heterozygosity excess tests, the proportion of multi-step mutations was set to 0.22, as well as the more commonly used 0.10 (i.e., *p*_*s*_ = 0.78 and 0.90, respectively); *∆*_*g*_ was set to 3.1. The *M*-ratio test also requires the input parameter pre-bottleneck *θ* (*θ* = 4*N*_*e*_*μ*; *N*_*e*_ = effective population size; *μ* = mutation rate) [73]. Given that pre-bottleneck *N*_*e*_ is unknown as is *μ*, a range of values for *θ* (0.2–20) was tested [48, 74]. If one assumes *μ* = 5.0 × 10^−4^, which is a commonly used microsatellite mutation rate [73, 75], these values correspond to pre-bottleneck *N*_*e*_ values: 100–10000 individuals. Observed *M* is considered significant and indicative of a population bottleneck if < 5% of simulated values fall below the observed *M* [73].

All bottleneck analyses were run using a data set including only adult red-bellied lemurs. In addition, because sex-biases in dispersal can influence the interpretation of bottleneck analyses (e.g., mask bottleneck signatures through introducing new alleles) [74, 76], all analyses were run using adult-female-only and adult-male-only data sets. Both data sets were used because behavioral observations in red-bellied lemurs suggest that both sexes disperse [51, 77], but it is unknown if there is sex bias in dispersal distance.

## Results

### Sex and M/L opsin genotyping

Sequences for exon 5 of the M/L opsin gene were obtained for 87 adult and immature red-bellied lemurs (*N*_female_ = 47, *N*_male_ = 40, *N*_adult_ = 58, *N*_immature_ = 29; *N*_X chromosomes_ = 134; see S3 Table). All individuals yielded codon ACC (amino acid = threonine) at site 285. Sex genotypes were consistent with color vision genotypes (i.e., males were not heterozygous) as well as sex assignments based on field observations. Given the final sample *N*_X chromosomes_ = 134, the frequency of the M allele is 0% and the L allele is 100%, indicating that all individuals are dichromats with the L opsin. This sample is more than sufficient to detect a color vision polymorphism present at a low (≥ 3%) frequency. That is, using cumulative binomial probability calculations, the probability of *not* detecting a rare (≥ 3%) allele given the sample size is < 0.05 (*p* < 0.001 for an allele at 5% frequency), which suggests the L opsin is effectively fixed in the population of red-bellied lemurs in RNP. *E*. *rubriventer* sequence data for exon 5 of the M/L opsin gene are available in FASTA format (S1 File).

### Microsatellite analysis

Of 59 adult individuals used in microsatellite genotyping analyses, 55 yielded confident genotypes at a minimum of 4 microsatellite loci and comprise the final data set (Table 1). Genotypes were 91% complete for 7 microsatellite loci (range 60–100% complete) across the 55 adult red-bellied lemurs (S4 Table). MICRO-CHECKER found no evidence for scoring errors, allelic dropout, or the presence of null alleles across each of the 7 loci. Of the 21 locus combinations, no combinations showed evidence for linkage after Bonferroni’s correction (*p* < 0.002). Summary statistics for all loci are presented in S4 Table. Across the 7 microsatellite loci, the mean number of alleles (*k*) was 5.86 (range 3 to 9). Mean allelic richness was 5.66 (range 3.00 to 8.31). Mean observed heterozygosity for the population was 0.69 (range 0.52 to 0.80) and mean expected heterozygosity was 0.70 (range 0.59 to 0.81). Goodness-of-fit tests showed no significant deviations from Hardy-Weinberg equilibrium for all loci.

**Table 1:** Sample used in microsatellite analysis includes adult individuals that yielded confident genotypes at a minimum of 4 microsatellite loci.

Site	*N*_groups_	*N*_males_	*N*_females_	*N*_individuals_
Talatakely	10	9	10	19
Sahamalaotra	4	2	3	5
Valohoaka	6	6	6	12
Vatoharanana	9	9	10	19
**Total**	**29**	**26**	**29**	**55**

### Genetic bottleneck analyses

#### Heterozygosity excess

Results of the Wilcoxon test for heterozygosity excess indicate that under TPM, the population of red-bellied lemurs in RNP exhibits significant excess heterozygosity compared to mutation-drift equilibrium. This was true when the proportion of multi-step mutations was set to 0.22 (*p* < 0.01) and the more conservative 0.10 (*p* < 0.05). Results were similar using an adult-female-only data set: *p* < 0.01 and *p* < 0.05 for proportions of multi-step mutations set to 0.22 and 0.10, respectively. Results approached significance using an adult-male-only data set: *p* = 0.055 when the proportion of multi-step mutations was set to 0.22 and 0.10.

#### *M*-ratio

*M*-ratio tests revealed that observed average *M* values in the population of red-bellied lemurs in RNP were high (0.94–0.97) and not significantly lower than expected under mutation-drift equilibrium (Table 2). This was the case for the combined male and female, female-only, and male-only data sets. Results also held under both scenarios for the proportion of multi-step mutations (0.22 and 0.10; *p*_*s*_ = 0.78 and 0.90, respectively).

**Table 2:** Results of *M*-ratio tests for the population of red-bellied lemurs in RNP. *M* = observed average *M* calculated across all loci for the combined male-female, female-only, and male-only data sets. The percentage of *M* values falling below observed *M* are given for both *p*_*s*_ = 0.78 (proportion of multi-step mutations = 0.22) and *p*_*s*_ = 0.90 (proportion of multi-step mutations = 0.10).

	Full data set			Female-only data set			Male-only data set		
Theta	*M*	% falling below *M*		*M*	% falling below *M*		*M*	% falling below *M*	
		*p*_*s*_ = 0.78	*p*_*s*_ = 0.90		*p*_*s*_ = 0.78	*p*_*s*_ = 0.90		*p*_*s*_ = 0.78	*p*_*s*_ = 0.90
0.2	0.97	90.97	67.71	0.91	74.74	43.98	0.97	90.63	67.54
1	0.97	98.52	88.77	0.91	92.50	67.54	0.97	98.38	89.94
2	0.97	99.62	95.43	0.91	98.21	83.34	0.97	99.78	96.19
10	0.97	100	99.88	0.91	99.95	99.08	0.97	100	99.98
20	0.97	100	100	0.91	100	99.88	0.97	100	100

## Discussion

**Fig 3:**
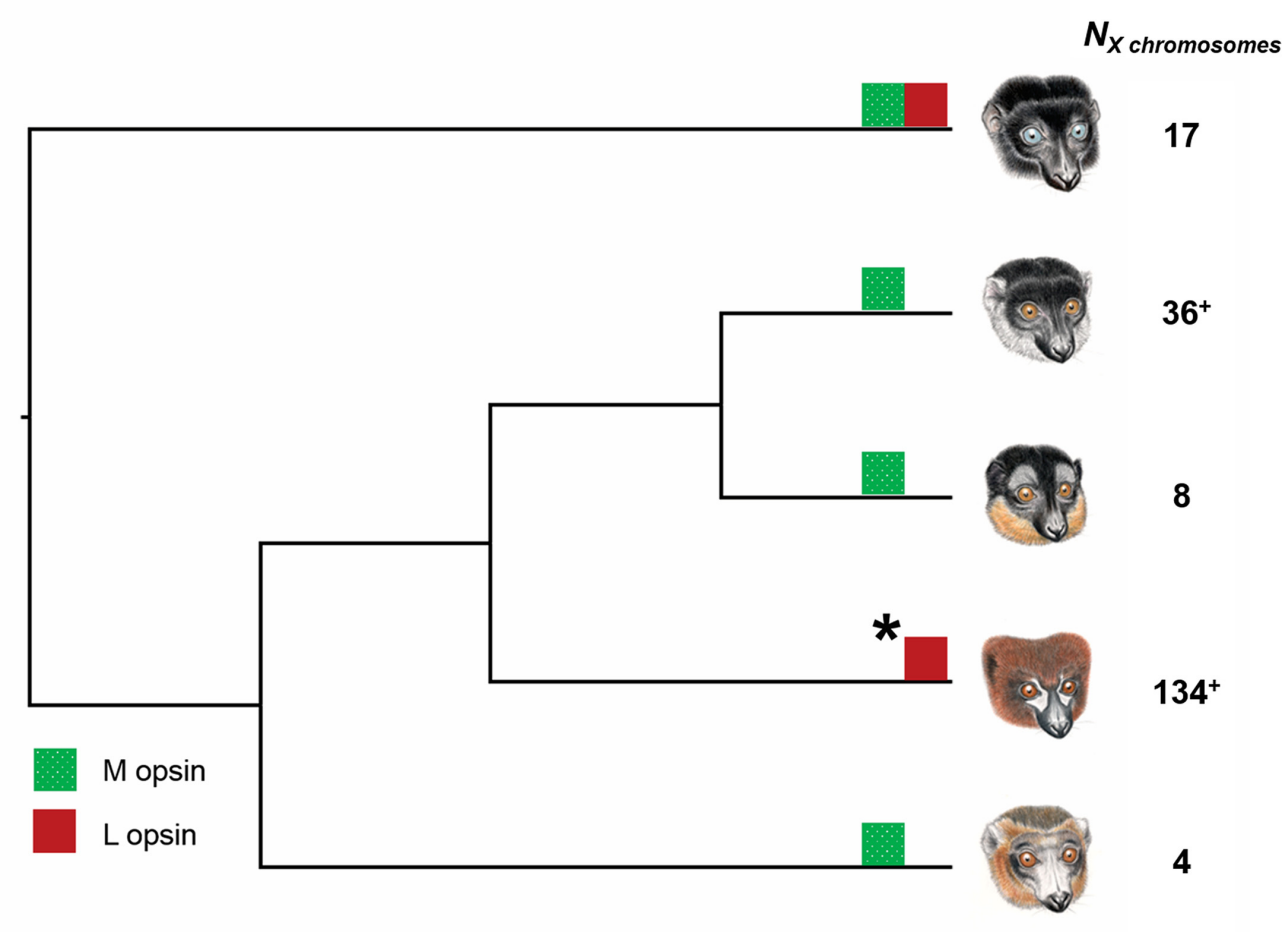
Phylogenetic distribution of opsin variation based on the current study (*) and published data. Numbers represent current sample sizes (X chromosomes). Those denoted with “^+^” are from wild populations. All other samples are from captive individuals. References (from the top): *E*. *flavifrons* [37], *E*. *fulvus* [43], *E*. *collaris* [36], *E*. *rubriventer* (this study), *E*. *mongoz* [11]. Phylogeny from [78]. *Eulemur* illustrations copyright 2015 Stephen D. Nash / Conservation International / IUCN SSC Primate Specialist Group and used in figure with permission from Stephen D. Nash.

Results of this study indicate that color vision in the population of *E*. *rubriventer* in Ranomafana National Park (RNP) is unique compared to other members of the genus *Eulemur* for which published data are available (Fig 3) [11, 36, 37, 43]. Specifically, all sequences for exon 5 of the M/L opsin gene in this population have the amino acid threonine at site 285 (L opsin: peak spectral sensitivity ~ 558 nm). The sample size used in this study is one of the most exhaustive samples of M/L opsins in lemurs to date and suggests that the L opsin is likely fixed in this population, although we cannot exclude the possibility that the M allele is present at a very low frequency (~2% or lower). Based on current sample sizes (Fig 3), other species of *Eulemur* appear to be either monomorphic for the M opsin (peak spectral sensitivity ~ 543 nm) or polymorphic [11, 36, 37, 43], which raises the question as to why our study population exhibits a different pattern of color vision than other *Eulemur*. Given the most recent *Eulemur* phylogeny [78] and using the principle of parsimony, it also appears that this population (and potentially *E*. *rubriventer* as a species) may have lost a color vision polymorphism (Fig 3). We note, however, that phylogenetic relationships of *Eulemur* remain unresolved (see also [79]), and we cannot discount the possibility that dichromacy for the L opsin is the ancestral *Eulemur* condition (e.g., see [80] on the ancestral primate color vision state).

It is, however, intriguing that color vision in *E*. *rubriventer*, as well as potentially other *Eulemur* species, might represent loss of color vision variation. Polymorphic color vision is thought to be maintained in many primate populations, particularly of New World monkeys, either through adaptive advantages of trichromatic color vision or advantages of trichromatic *and* dichromatic color vision (i.e., balancing selection) [1, 9, 12]. Although empirical support for such advantages remains limited [23, 25–27, 29, 32].

While not discounting the potential for selective processes, such as disruptive and/or directional selection, including a selective sweep, to account for *loss* of color vision variation [81], results of this study suggest that nonadaptive processes may also play a role. Specifically, heterozygosity excess tests indicate that the population of red-bellied lemurs in RNP may have experienced a genetic bottleneck. Interestingly, heterozygosity excess has also been documented in populations of *E*. *collaris* [82], a species that is presently considered to be monomorphic for the M opsin (although this is based on captive individuals [36]). Furthermore, evidence for bottlenecks has been found across a number of lemur species, which might be related to anthropogenic disturbances throughout Madagascar [44–48]. Taken together, such results suggest genetic bottlenecks may be widespread among wild lemur populations. Given that the impact of genetic drift increases in small populations, a recent population crash could result in loss of allelic variation, and this can occur even in the presence of positive selection [81]. That is, loss of color vision variation could occur in bottlenecked populations under multiple scenarios including relaxed selection to maintain color vision variation, selection favoring one opsin allele over another, and even selection favoring allelic variation.

While our results suggest that a genetic bottleneck might have occurred in our study population, it is important to note that these results are not unequivocal; heterozygosity excess tests indicate a potential genetic bottleneck, but *M*- ratio tests do not. Increasing the number of loci might help resolve inconsistent results by increasing power [71]. Nonetheless, a similar pattern (significant heterozygosity excess coupled with high *M*-ratios) has been identified in other vertebrate species (e.g., ornate box turtles: [83]; northern spotted owls: [84]; Siberian tigers: [85]). The opposite pattern, in which *M*-ratio tests but not heterozygosity excess tests show signatures of bottlenecks, has also been observed (e.g., tiger salamanders: [86]; copperbelly water snakes: [87]; elk: [88]; bottlenose dolphins: [89]). One proposed explanation for ambiguous results suggests that different tests are better able to detect bottlenecks that vary in timing/duration and/or severity [72]. For example, heterozygosity excess tests may be better at detecting recent or less severe bottlenecks, while the *M*-ratio test may be better able to detect bottlenecks in populations that have had some recovery time, or in those that have experienced longer-term bottlenecks (i.e., multiple generations) [72]. Accordingly, mixed results are not necessarily inconsistent with a genetic bottleneck, and, in fact, a more recent bottleneck would accord well with the recent large-scale forest destruction that has occurred across the eastern rainforests, including the Ranomafana region [90]. However, these analyses do not date or quantify population bottlenecks. Additional analyses designed to evaluate the timing and scale of population decline, such as the Bayesian method of [91], may help clarify whether and to what extent a genetic bottleneck occurred in this population.

It should also be acknowledged that multiple confounding factors can produce spurious bottleneck signatures, one of which is population substructure (F_st_ ≥ 0.1) [92]. All samples used in this study were collected from multiple groups at four sites located within 8 km of each other and appear to be in migratory contact, which is supported by low pairwise F_st_ values (F_st_< 0.1; S5 Table). However, genetic differentiation between sites was significant in some cases, with the greatest differentiation occurring between Sahamalaotra and two sites located in the southern parcel (Fig 2). If we remove Sahamalaotra from the analyses, however, significant heterozygosity excess remains under most conditions (S1 Text).

That said, there are additional factors that might result in type I errors, such as sampling scheme and immigration. Chikhi et al. [92] found that in highly structured populations, false bottleneck signatures were more likely to be obtained when sampling from a single “deme”. In order to counter this, they suggested sampling from multiple demes [92]. Although our study used samples collected from multiple localities and groups, likely minimizing this potential effect, it is possible that the population of *E*. *rubriventer* in RNP exhibits larger-scale structure, with the samples used here representing a single deme.

Finally, one of the assumptions of the heterozygosity excess test is the absence of migration between populations [67]. This assumption is often violated, and while low-level immigration likely masks a bottleneck effect [74], high levels of immigration can actually mimic a population bottleneck [93]. Although RNP is disconnected from forest tracts to the north [94], a narrow corridor to larger tracts of forest to the south remained as of 2000 [90]. Whether or not this physical connectivity actually facilitates migration is unknown, but if there is a high level of immigration from southern populations, this could potentially result in a bottleneck signal without population collapse. At the same time, a spurious bottleneck effect can be obtained when once-connected populations become completely disconnected, without actual population collapse [95]. Such a scenario may be applicable to RNP, which was historically connected to larger and continuous tracts of forest [90, 94]. Future studies incorporating simulations, as well as additional data from RNP and other populations of red-bellied lemurs, will help tease apart the potential effects of population collapse, population structure, and migration on the excess heterozygosity observed in this study.

This study has identified *E*. *rubriventer* to be unique among other *Eulemur* in being dichromatic with the L opsin based on data from a wild population that might have experienced a recent genetic bottleneck. Sampling additional populations throughout *E*. *rubriventer*’s range will be important to determine if dichromatic color vision with the L opsin characterizes the species or is specific to particular populations. Understanding opsin variation within the larger context of genomic variation [32] will also help clarify the roles of selection and drift in wild lemur populations. Such studies are increasingly feasible as methods advance for conducting genomic-level variation analyses using non-invasive samples [96].

## Supporting Information

S1 File *Eulemur rubriventer* M/L opsin gene, exon 5 and partial cds (FASTA format). (FASTA)

S1 Table Number of individual red-bellied lemurs for which fecal samples were collected in RNP. Samples were collected between January 2012 and May 2013.
(PDF)

S2 Table Characteristics of 7 variable microsatellite loci for *E*. *rubriventer* that were used in this study. Locus names, primer sequences, and repeat motifs are from [60]. Size ranges represent size ranges obtained in this study. Annealing temperatures (*T*) were modified when necessary from [60].
(PDF)

S3 Table *E*. *rubriventer* samples from RNP that were genotyped at exon 5 of the M/L opsin gene. (PDF)

S4 Table Summary statistics for 7 microsatellite loci (*N* = 55 individuals) for the red-bellied lemur population in RNP. (PDF)

S5 Table Pairwise F_st_ values for each sample locality within RNP. F_st_ values are above the diagonal and *p* values are below. *p* values that were below the Bonferroni-corrected significance value of 0.05 (*p* < 0.008) are in bold.
(PDF)

S1 Text Results of heterozygosity excess tests excluding samples from Sahamalaotra. (PDF)
