# Supplementary material for: Considering the Influence of Nonadaptive Evolution on Primate Color Vision
Source: PLoS One. 2016 Mar 9;11(3):e0149664. doi: 10.1371/journal.pone.0149664 (PMC4784951; doi:10.1371/journal.pone.0149664)
Supplement: S1 Table — Samples were collected between January 2012 and May 2013. (PDF) [file pone.0149664.s002.pdf]

S1 Table. Number of individual red-bellied lemurs for which fecal samples were collected in RNP. Samples were collected between January 2011 and May 2013.

**Site**	N_{groups}	$N_{adult\ males}$	$N_{adult\ females}$	$N_{immature\ females}$	$N_{immature\ males}$	$N_{individuals}$
Ambatolahy dimy	4	4	3	1	1	9
Sahamalaotra	4	3	4	2	2	11
Sakaroa	11	9	10	4	3	26
Talatakely	11	11	13	9	4	37
Valohoaka	10	10	10	9	9	38
Vatoharanana	12	12	12	5	7	36
**Total**	**52**	**49**	**52**	**30**	**26**	**157**
