# Supplementary material for: Considering the Influence of Nonadaptive Evolution on Primate Color Vision
Source: PLoS One. 2016 Mar 9;11(3):e0149664. doi: 10.1371/journal.pone.0149664 (PMC4784951; doi:10.1371/journal.pone.0149664)
Supplement: S2 Table — Locus names, primer sequences, and repeat motifs are from [60]. Size ranges represent size ranges obtained in this study. Annealing temperatures (T) were modified when necessary from [60]. (PDF) [file pone.0149664.s003.pdf]

S2 Table. Characteristics of 7 variable microsatellite loci for *E. rubriventer* that were used in this study. Locus names, primer sequences, and repeat motifs are from [60]. Size ranges represent size ranges obtained in this study. Annealing temperatures (*T*) were modified when necessary from [60].

**Locus**	**Primer sequence**	**Repeat Motif**	**Size range (bp)**	***T* (°C)**
44HDZ005	F: GAGCCCAGAGTGCCTTTG<br>R: GAGATTAGAGAAGTATGTGTGTTTGG	$(GT)_{17}$	161-169	54
44HDZ011	F: TGTGGATTCAGCATTTGGC<br>R: TCTGTCAGGGATTTGCGAG	$(CA)_{16}$	162-182	56
44HDZ035	F: ACCTCACCTCGCCTAGTCC<br>R: TGCCTCTCGTGTTTGGTTC	$(AC)_{15}$	148-160	54
44HDZ119	F: TGGTTTTGCCACAAGTTATGTC<br>R: TGAAGCCATCTAAGGAGGTTG	$(CA)_{12}$	158-162	60
44HDZ124	F: TACACCCCCTCCCCCAA<br>R: GGCAAGTCTTTTGTCTAATGGAA	$(CA)_{16}$	132-140	54
44HDZ193	F: TCTGTGTAAGAAAAATGGGGAC<br>R: AGCCAGGAACTGTGGACG	$(CA)_{14}$	171-185	54
44HDZ287	F: GTTTCCCCTACCAAGCTGC<br>R: ATGGAAAAGGAGGTAGCAATG	$(CA)_{23}$	173-179	57
