# Supplementary material for: Considering the Influence of Nonadaptive Evolution on Primate Color Vision
Source: PLoS One. 2016 Mar 9;11(3):e0149664. doi: 10.1371/journal.pone.0149664 (PMC4784951; doi:10.1371/journal.pone.0149664)
Supplement: S3 Table — (PDF) [file pone.0149664.s004.pdf]

S3 Table. *E. rubriventer* samples from RNP that were genotyped at exon 5 of the M/L opsin gene.

**Site**	N_{groups}	$N_{adult\ males}$	$N_{adult\ females}$	$N_{immature\ males}$	$N_{immature\ females}$	$N_{X\ chromosomes}$
Ambatolahy dimy	1	1	0	0	0	1
Sahamalaotra	4	2	4	0	1	12
Sakaroa	6	2	4	1	2	15
Talatakely	9	9	8	2	4	35
Valohoaka	9	7	7	5	7	40
Vatoharanana	7	7	7	4	3	31
**Total**	**36**	**28**	**30**	**12**	**17**	**134**
