# Supplementary material for: Considering the Influence of Nonadaptive Evolution on Primate Color Vision
Source: PLoS One. 2016 Mar 9;11(3):e0149664. doi: 10.1371/journal.pone.0149664 (PMC4784951; doi:10.1371/journal.pone.0149664)
Supplement: S4 Table — (PDF) [file pone.0149664.s005.pdf]

S4 Table. Summary statistics for 7 microsatellite loci (*N* = 55 individuals) for the red-bellied lemur population in RNP.

**Locus**	***k***	**AR**	**N**	**H_{Obs}**	**H_{Exp}**	**PIC**	**NE_{1P}**	**NE_{2P}**	**NE_{PP}**	**NE_{ID}**	**NE_{SibID}**	***p***	**F (Null)**
44HDZ005	5	4.938	55	0.764	0.689	0.626	0.737	0.573	0.397	0.157	0.448	0.178	-0.0626
44HDZ011	9	8.309	47	0.766	0.800	0.761	0.583	0.405	0.222	0.074	0.373	0.737	0.0186
44HDZ035	7	6.537	55	0.745	0.763	0.719	0.640	0.461	0.274	0.096	0.396	0.434	0.0080
44HDZ119	3	3.000	33	0.576	0.597	0.522	0.827	0.682	0.531	0.236	0.515	0.897	0.0026
44HDZ124	5	4.930	53	0.642	0.634	0.564	0.786	0.634	0.465	0.202	0.487	0.877	-0.0087
44HDZ193	8	7.938	55	0.800	0.810	0.782	0.545	0.367	0.177	0.059	0.363	0.878	0.0004
44HDZ287	4	3.989	52	0.519	0.586	0.496	0.826	0.702	0.556	0.261	0.525	0.487	0.0604

**Statistics for combined loci**	
Mean number of alleles	5.857
Mean H_{Exp}	0.697
Mean PIC	0.639
Combined NE_{1P}	0.080
Combined NE_{2P}	0.119
Combined NE_{PP}	5.88×10^{-4}
Combined NE_{ID}	8.30×10^{-7}
Combined NE_{SibID}	3.16×10^{-3}

k = number of alleles, AR = allelic richness, N = number of individuals genotyped, H_{Obs} = observed heterozygosity, H_{Exp} = expected heterozygosity under Hardy-Weinberg equilibrium, PIC = polymorphic information content, NE_{1P} = non-exclusion probability (first parent), NE_{2P} = non-exclusion probability (second parent), NE_{PP} = non-exclusion probability (parent-pair), NE_{ID} = non-exclusion probability (identity), NE_{SibID} = non-exclusion probability (sibling identity)
