# Supplementary material for: Considering the Influence of Nonadaptive Evolution on Primate Color Vision
Source: PLoS One. 2016 Mar 9;11(3):e0149664. doi: 10.1371/journal.pone.0149664 (PMC4784951; doi:10.1371/journal.pone.0149664)
Supplement: S5 Table — Fst values are above the diagonal and p values are below. p values that were below the Bonferroni-corrected significance value of 0.05 (p < 0.008) are in bold. (PDF) [file pone.0149664.s006.pdf]

S5 Table. Pairwise F_{st} values for each sample locality within RNP. F_{st} values are above the diagonal and *p* values are below. *p* values that were below the Bonferroni-corrected significance value of 0.05 ($p < 0.008$) are in bold.

	Sahamalaotra	Talatakely	Valohoaka	Vatoharanana
Sahamalaotra	---	0.066	0.081	0.099
Talatakely	0.009	---	0.022	-0.008
Valohoaka	**0.002**	0.053	---	0.047
Vatoharanana	**0.001**	0.813	**0.003**	---
