# Supplementary material for: Considering the Influence of Nonadaptive Evolution on Primate Color Vision
Source: PLoS One. 2016 Mar 9;11(3):e0149664. doi: 10.1371/journal.pone.0149664 (PMC4784951; doi:10.1371/journal.pone.0149664)
Supplement: S1 Text — (PDF) [file pone.0149664.s007.pdf]

S1 Text. Results of heterozygosity excess tests excluding samples from Sahamalaotra.

Heterozygosity excess tests using the full data set but excluding samples from Sahamalaotra ($N = 50$ individuals) yielded similar results as those including Sahamalaotra. There was significant heterozygosity excess under both multi-step mutation assumptions: $p < 0.01$ (0.22 multi-step mutations) and $p < 0.05$ (0.10 multi-step mutations). However, when only females were analyzed in this sample ($N = 26$), heterozygosity excess was significant when the proportion of multi-step mutations was set to 0.22 only ($p < 0.05$; $p = 0.148$ with 0.10 multi-step mutations). Results were similar when only males were analyzed. Heterozygosity excess was significant when the proportion of multi-step mutations was set to 0.22 only ($p < 0.05$; $p = 0.344$ with 0.10 multi-step mutations).
